# Back to the future: The advantage of studying key events in human evolution using a new high resolution radiocarbon method

**DOI:** 10.1371/journal.pone.0280598

**Published:** 2023-02-15

**Authors:** Sahra Talamo, Bernd Kromer, Michael P. Richards, Lukas Wacker

**Affiliations:** 1 Department of Chemistry G. Ciamician, Alma Mater Studiorum, University of Bologna, Bologna, Italy; 2 Institute for Environmental Physics, University of Heidelberg, Heidelberg, Germany; 3 Department of Archaeology, Simon Fraser University, Burnaby, B.C., Canada; 4 Laboratory for Ion Beam Physics, ETH Zurich, Zurich, Switzerland; University of California Santa Cruz, UNITED STATES

## Abstract

Radiocarbon dating is the most widely applied dating method in archaeology, especially in human evolution studies, where it is used to determine the chronology of key events, such as the replacement of Neanderthals by modern humans in Europe. However, the method does not always provide precise and accurate enough ages to understand the important processes of human evolution. Here we review the newest method developments in radiocarbon dating (‘Radiocarbon 3.0’), which can lead us to much better chronologies and understanding of the major events in recent human evolution. As an example, we apply these new methods to discuss the dating of the important Palaeolithic site of Bacho Kiro (Bulgaria).

## Introduction

Precise absolute dating of human bones or bone objects is crucial for understanding the chronological timing of cultural events in Palaeolithic Archaeology. In recent decades, many scientific studies have been published proposing the “earliest” or the “latest” finds in terms of dispersal, interaction, and demise of archaic humans [1–7], extinctions of animals [8–10], the emergence of innovative cultural behaviours [11–13], and interplays between hominins and climatic fluctuations [14–19]. The question arises, however, about how confident we really are about emerging hypotheses based on statistically debatable age models. Do we really have such a high chronological resolution to say that those fossils or finds are the "oldest" or "youngest"? Do we have a sufficient number of archaeological sites with robust and high-resolution chronologies to corroborate our hypothesis? If we look at the time period of the replacement of Neanderthals by *Homo sapiens* in Europe (The so-called Middle to Upper Palaeolithic transition), the resolution of most of the radiocarbon dates is so low that we could suggest a chronological overlapping between the two species across different regions of *c*. 2.000 years. In recent years, new dating methodologies (e.g., Optically Stimulated Luminescence (OSL), Electron Spin Resonance (ESR), or Hydroxyproline ^14^C (HYP)) [6, 20–22] have been used with even larger standard deviation errors on the dates that increase, rather than reduce, the uncertainties on the interpretation of our archaeological record. Thus far, palaeogenetics has been the only research field that confirmed the interbreeding between Neanderthals, Denisovans, and *Homo sapiens* [23–25], whereas, from cultural and chronological perspectives, we are still unable to provide a clear chronology for this scenario. How could we do better and improve the chronometric data of the late Middle and Upper Palaeolithic? The major challenge of this scientific dare is related to a) the radiocarbon method and b) the quality of the archaeological record.

Radiocarbon (^14^C) is considered the most important and universal dating method in archaeology for several reasons: [1] The dating range of the past 55,000 years covers an essential part of the later stage of human evolution, especially the transition from Neanderthal to *Homo sapiens* in Eurasia, [2] Carbon is a ubiquitous element in the environment and [3] organic components recovered in archaeological sites can often be linked directly to human activities. Numerous ‘Radiocarbon Revolutions’ have occurred, from the first application of radiocarbon dating [26, 27] to the construction of a calibration curve [28–32], the development of accelerator mass spectrometers (AMS) to measure ^14^C in small samples [33–35], and the wide-spread use of Bayesian statistics to analyse and interpret radiocarbon data linked to ‘prior’ information [36, 37].

Today the radiocarbon dating method is even more intertwined with the field of archaeology, and there are new improvements in the method, which we call ‘Radiocarbon 3.0’, due to the updated radiocarbon pretreatment methods [34, 38], the latest AMS instrumental advances (e.g., increasingly precise error ranges [13, 35, 39]), and the latest improvement of the calibration curve, IntCal20 [31, 40, 41]. However, it is not always straightforward to make use of these substantial advances because when organic material is used to obtain a ^14^C date, several factors need to be considered.

First of all, we need to retrieve enough organic material for dating. This involves the condition of the organic material itself that is submitted for analysis, e.g., thick cortical animal bones with human modification traces are selected to obtain good quality collagen [38, 42]. Once the organic material is extracted, it is converted to a graphite target or the CO_2_ is directly injected to obtain a precise ^14^C age with AMS.

Secondly, ^14^C measurements are always accompanied by a measure of uncertainty, which is the statistical error range produced by the AMS counting technique. The better this error range is defined and obtained, the more accurate the final age calibration process will be. The recent advances in the AMS instruments lead to high efficiency combined with low and stable backgrounds, which is key to getting low uncertainties.

Thirdly, an accurate calendar age calibration of the ^14^C age is required since the ^14^C signature of a living organism (as the starting point of the ^14^C decay) varied in the past [43]. Therefore, ^14^C ages must be calibrated against ^14^C data sets constructed from independently dated archives storing the past atmospheric ^14^C signature. Calibration data sets were created and internationally accepted since 1993 [44]–called IntCal. The most recent one is IntCal20 [31].

Ideal for calibration are dendrochronologically dated tree-ring chronologies because ^14^C in tree rings reflects the atmospheric ^14^C level of the respective year. In the IntCal20 calendar age dated tree-ring chronologies are available back to 14,200 years cal BP (calibrated years Before Present, 1950 AD), with decadal to the annual resolution of ^14^C dates and statistical errors of 15 to 30 years (1σ) [45]. Before this time, back to 55,000 cal BP, macrofossils from laminated lake sediments and independently dated carbon archives linked only indirectly to the atmosphere were used. Examples are speleothems and marine sediments. The resolution and accuracy of ^14^C data from these archives are much lower than those from tree rings. For instance, in the age range of around 42,000 cal BP, one date every 120 to 250 years has been measured, with errors of 250 to 350 years.

Though trees of glacial ages have been found, and tree-ring chronologies have been constructed [46, 47], they were not considered useful for calibration, because they are not linked to absolutely dated chronologies. Only recently, a method to link ^14^C variations to similar signals in cosmogenic isotopes, especially Beryllium-10 (^10^Be), has been developed [48, 49]. However, in IntCal20, the ^10^Be links were not used, and these floating tree-rings were ^14^C wiggle matched, providing segments of calibration intervals of high temporal resolution and high precision [48]. Moreover, recent improvements have been shown by adding a section of floating tree-ring chronologies covering the period between 44,000 to 41,000 cal BP [18]. This new ^14^C dataset of floating subfossil Kauri trees from New Zealand has a temporal resolution of 40 years and errors of 120 to 220 years. Those ^14^C series can now be combined with the speleothem-derived radiocarbon dataset reported from Hulu Cave [50], adding more structural details to the so far smooth calibration curve during this period of time. Additional precise ^14^C datasets of floating tree-ring chronologies are currently under review, and they will be placed soon for different time periods in the Glacial (https://site.unibo.it/resolution-erc/en).

Finally, to securely evaluate the relationship between dated samples and their archaeological context, models using Bayesian statistics are crucial. The accuracy of these models strongly depends on the kind of data that are introduced: e.g., the number of samples dated, the ‘prior’ information (e.g., stratigraphy), and the error range of the ^14^C dates [51]. Moreover, a precise chronology is also strictly related to the quality and preservation of the archaeological record. A broad consensus suggests that a defined archaeological level is often the result of successive episodes of activities and depositions over variable periods of time and compressed into a single layer or surface [52, 53]. Therefore, stratigraphically defined bone and lithic assemblages are not always synchronous but temporally distinct [54, 55]. Refitting of pieces of bones and lithic artefacts has been useful for dissecting these palimpsests showing the temporal relation between the archaeological accumulations [42, 54, 56, 57]. In this manner, the conjoining of pieces of bone and artefact allows avoiding misconceptions on the interpretation of the archaeological level and inferring an “average behaviour” rather than an inter-assemblage variability [54]. In this perspective, the identification of these palimpsests could also be beneficial for the site chronology, in which radiocarbon 3.0 could at least distinguish in smaller temporal ranges the duration of an archaeological layer.

The combination of all these factors increases the complexity in the nature of the data, and this means that archaeologists need to have a critical understanding of what kind of ^14^C results they take into account in order to build an accurate and robust chronology for an archaeological site, especially in the Middle to Upper Palaeolithic (MUP).

This paper investigates the benefits of using radiocarbon 3.0 in an archaeological site where all the above-mentioned criteria are evaluated. In this exercise, we use the robust chronology of Bacho Kiro Cave (Bulgaria), the only archaeological site with more than 21 high-resolution radiocarbon dates for a single layer and less than 400 years ^14^C error range for a date around 42,000 years. We combine this chronometric data with the new section of the floating tree-ring chronology of Kauri trees [18] in OxCal.

The results of our study document how the use of the high-level radiocarbon method can have an impact on the detailed study of an archaeological site and has a great potential to infer a more realistic chronology of the Bacho Kiro Initial Upper Palaeolithic (IUP) using two or three temporally distinct human occupations at the site and put the new information within the context of different climatic events. Overall, our new data leads to a more accurate discussion on the early dispersal of *Homo sapiens* in Europe.

## Materials and methods

### Material

Here we explore the radiocarbon dates from layers J and I of the Niche of Bacho Kiro site published in [35], as well as the four direct dates of *Homo sapiens* fossils, found only in the Niche N1-I layer. Four more Neanderthal bones were added to this study in order to investigate the differences between small and wider ^14^C error ranges when we calibrate radiocarbon ages. Two Neanderthals are from the Vindija cave in Croatia, which were pretreated at MPI-EVA (Max Planck Institute for Evolutionary Anthropology) following the procedures published in [38, 58]. The extracted collagen was sent to the Oxford Radiocarbon Accelerator Unit (ORAU) for the graphitization and AMS dates (OxA-V-2291-19 and OxA-V-2291-18); one was published in [59], and one is published in this study.

One Neanderthal from the same site was pretreated with the HYP ^14^C method by ORAU (OxA-X-2717-11) and published in [22]. The fourth direct date of Neanderthal was pretreated in the same procedure as the one described above using the HYP ^14^C method (OxA-38322) and published in [6]. All samples are listed in Table 1.

**Table 1:** Radiocarbon ages of the directly dates of 4 *Homo sapiens* from Bacho Kiro, 4 Neanderthals from Fond-de Forêt and Vindija. The respective calibrated results using both IntCa20 [31] in OxCal4.4 [58] and IntCal 20+Kauri section.

		Unmodelled (BP) IntCal20				Unmodelled (BP) IntCal20+Kauri			
Site, Region, References	AMS Code (^14^C Age±1σ Err)	from	to	from	to	from	to	from	to
		***68*.*3%***		***95*.*4%***		***68*.*3%***		***95*.*4%***	
Bacho Kiro, Bulgaria Fewlass et al. 2020	*Homo sapiens* ETH-86772 HS (42450;510)	45430	44640	45930	44420	45440	44640	45950	43950
Bacho Kiro, Bulgaria Fewlass et al. 2020	*Homo sapiens* ETH-86770 HS (41850;480)	45010	44320	45550	43940	45040	43940	45470	43520
Bacho Kiro, Bulgaria Fewlass et al. 2020	*Homo sapiens* ETH-86771 HS (40600;420)	44080	43240	44400	42990	44190	43130	44420	42950
Bacho Kiro, Bulgaria Fewlass et al. 2020	*Homo sapiens* ETH-86769 HS (39750;380)	43240	42700	43930	42580	43380	42700	44020	42400
Vindija 33.39, Croatia In this study	Neanderthal OxA-V-2291-19-MPI (44500;600)	47430	46090	48210	45710	47430	46090	48210	45710
Vindija 33.26, Croatia Green, et al.2010	Neanderthal OxA-V-2291-18-MPI (44450;550)	47330	46080	48100	45760	47330	46080	48100	45760
Vindija 33.19, Croatia Devièse et al, 2017	Neanderthal OxA-X-2717-11-HYP (44300;1200)	47980	45550	49930	44690	47980	45550	49940	44690
Fond-de Forêt, Belgium Devièse et al, 2021	Neanderthal OxA-38322 (39500;1100)	43880	42430	44860	42070	44040	42310	44800	42050

### Calibration curve

For calibration of the ^14^C ages, we use a modified version of IntCal20 [31] in the OxCal 4.4 program [60]. In our version, the data of IntCal20 [31] are replaced by the original Kauri ^14^C data and uncertainties [18] (no splines or smoothing) in the range of the combined Kauri chronologies (43977–41116 cal BP), here called IntCal20+Kauri. The Kauri data were not yet available at the time of the construction of IntCal20.

**Fig 1:**
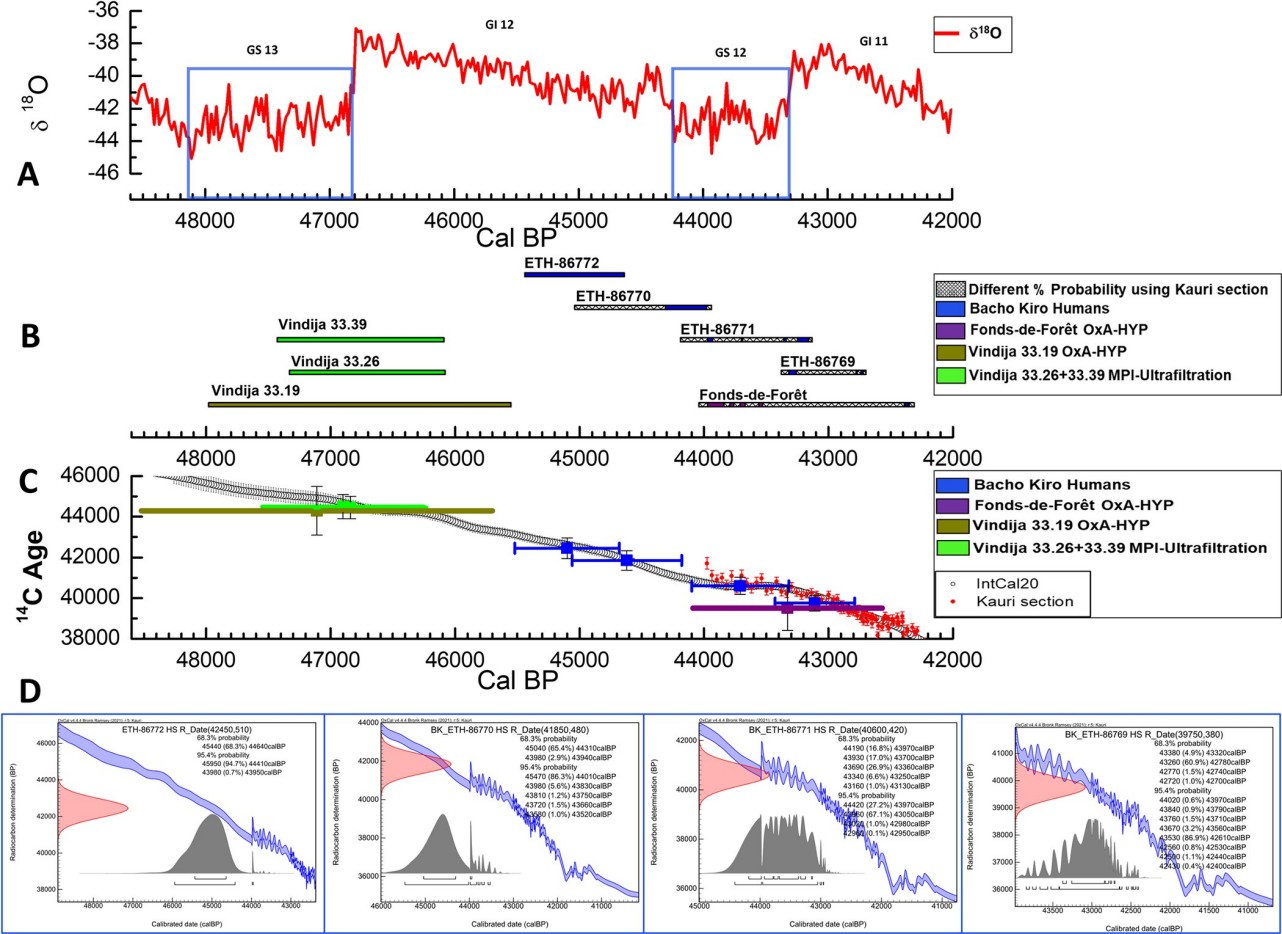
^14^C error range and Kauri floating curve. A) the red curve represents the climatic events using the data of δ^18^O, and the terminology of the interstadial (GI) and stadial (GS) events of the Greenland ice cores published [59], in blue squares indicate the cold phases of GS13 and GS12; in the black square on the right the entire sequence of the NGRIP from 49,000 to 42,000 cal BP. B) the calibrated ranges obtained by the direct radiocarbon dates on 4 *Homo sapiens* (Bacho Kiro) and 4 Neanderthals (Vindija and Fonds-de-Forêt) fossils [6, 22, 35, 62]. C) the mean with the relative 1σ error produced by OxCal for the human fossils dated on the IntCal20 calibration curve [31] in black and on Kauri floating section [18] in red. D) the calibrated ranges using IntCal+Kauri obtained using the OxCal 4.4 program [58].

**Fig 2:**
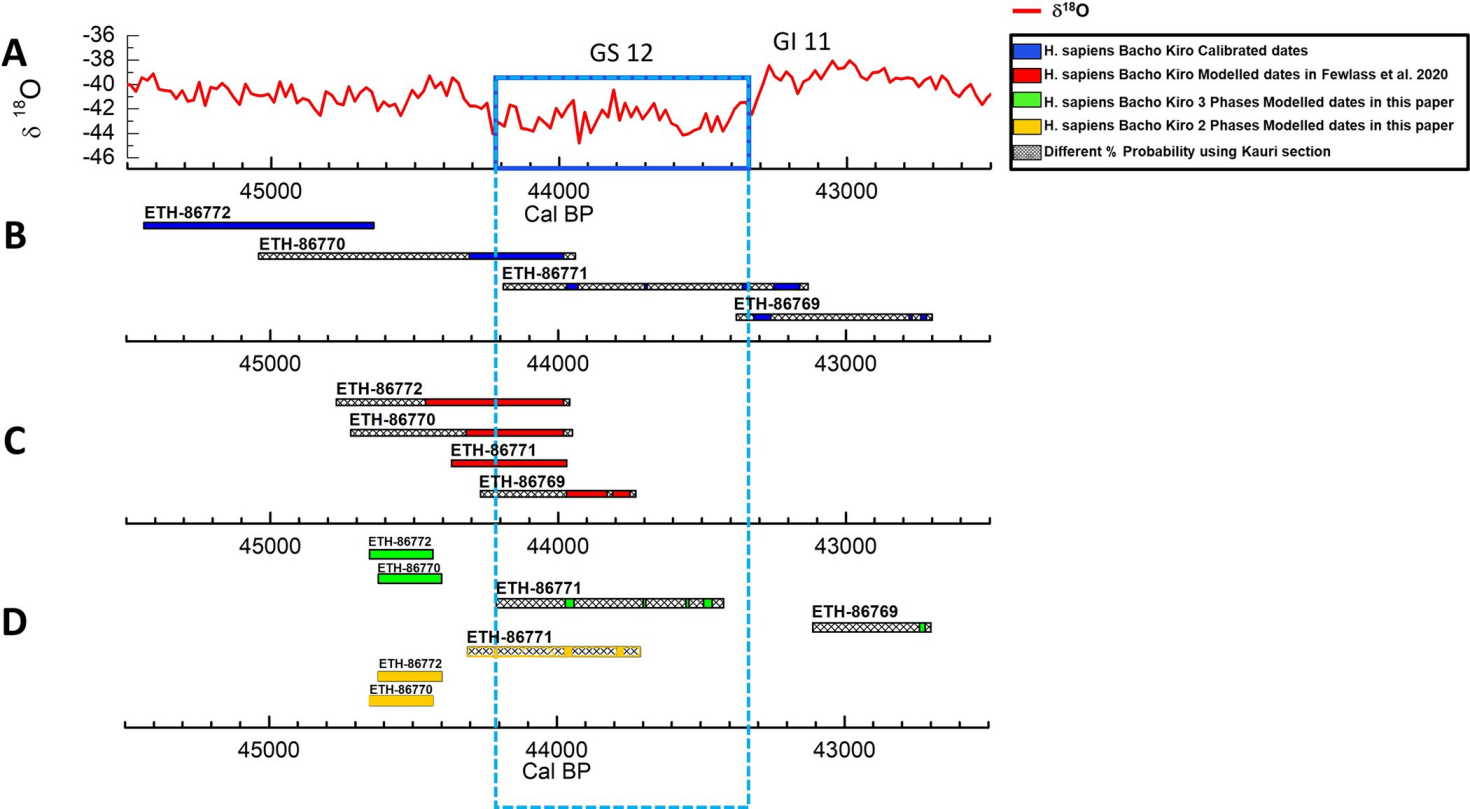
Unmodelled vs Modelled of the direct dates of Bacho Kiro *Homo sapiens*. A) the NGRIP δ^18^O curve spanning 45,500 to 42,500 cal BP. B) the calibrated ranges of the 4 Bacho Kiro *Homo sapiens* directly dated. C) the calibrated ranges obtained using OxCal model in [35] of the 4 Bacho Kiro *Homo sapiens*. D) the calibrated ranges of the 4 Bacho Kiro *Homo sapiens* were obtained using the 3 phases model (green) and 2 phases model (yellow). For all the bars, the hatched age ranges show differences using the IntCal20+Kauri curve. The blue rectangle highlights the GS12 cold climate phase.

The calibrated dates using the IntCal20+Kauri curve have wider ranges than the calibrated dates using only IntCal20 (S1 File). This situation is due to the fact that the new piece of the curve introduces many more wiggles and plateaux compared with the smooth IntCal20. This means that one single date can have multiple probability ranges (Figs 1D and 2), compared with IntCal20, which results in only one range with 68.3% probability.

**Fig 3:**
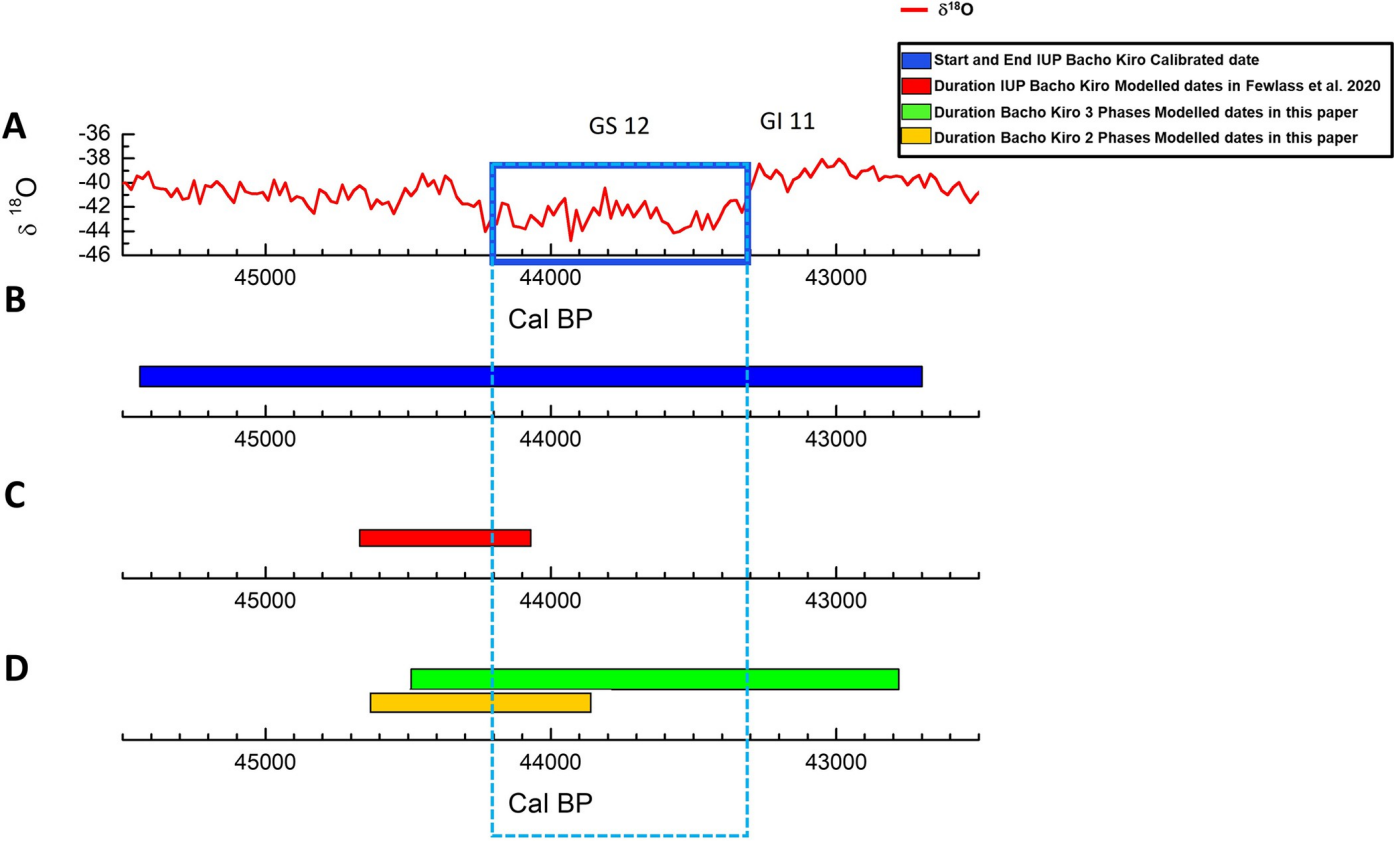
Unmodelled vs Modelled IUP boundaries of Bacho Kiro site. A) the NGRIP δ^18^O curve spanning 45,500 to 42,500 cal BP. B) the IUP duration obtained by the simple calibration dates of the N1-I layer. C) the IUP duration obtained by the OxCal model in [35] of the N1-I layer. D) the IUP duration obtained by the 3 phases model (green) and 2 phases model (yellow). The blue rectangle highlights the GS12 cold climate phase.

We are aware that there is a 1 in 20 chance that the true date falls outside of 95.4% and a 1 in 3 chance that it falls outside of the 68.3% age range; hence the 95.4% is less likely to be wrong. However, given the generally high ^14^C age error range (> 400 years, up to 1100 years) produced by different ^14^C laboratories in the time period considered here, the comparison of the centre probability appears more illuminative (e.g., Fig 1C). Moreover, as it is shown in S1 File, Column AD, the mean calibrated interval of 95.4% probability is almost 3 times wider than the one of 68.3%, which, in our study, will aggravate detailed comparisons. Hence the ^14^C age and calibrated results discussed here are shown in Figs 1B, 2 and 3 as 1-sigma (68.3% probability) and 2-sigma (95.4% probability) ranges are considered in the S1 File and in S1 and S2 Figs.

All our results are compared to the climatic events using the data of δ^18^O and the terminology of the interstadial (GI) and stadial (GS) events of the Greenland ice cores published in [61]. The variation of the climate parameters (e.g., temperature, precipitation) appears of similar magnitude in Southeastern Europe compared to Greenland [62].

### OxCal model

We note that based on radiocarbon dates, layer N1-I is composed of three different clusters of dates: one around 42,000 to 41,000 ^14^C BP, one around 40,000 ^14^C BP and one around 39,000 to 38,000 ^14^C BP. The mean ^14^C age of these three groups was compared using the t-test in Excel (two-tailed distribution, equal variance). The *p*-value of group 1 vs. group 3 is 6.8E-8, critical t-value = 3.18, t-value = 5.89, so the difference is highly significant. The same result is obtained for group 1 vs. group 2, with p = 3.2E-05, critical t-value = 2.22, and t-value = 7.11. Even for group 2 vs. group 3, the p-value is 0.044, critical t-value 3.18, and t-value 3.32, hence significant (Table 2).

**Table 2:** T-test values of the 3 different groups of the dates in layer N1-I from [35]. The dates in red are the *Homo sapiens* directly dated.

1^st^ Group			2^nd^ Group			3^rd^ Group		
AMS Code	^14^C Age	1σ Err	AMS Code	^14^C Age	1σ Err	AMS Code	^14^C Age	1σ Err
ETH-71318	41080	260	ETH-86783	40340	280	ETH-86769	39750	380
ETH-71327	41170	260	ETH-86771	40600	420	ETH-86782	39710	260
ETH-71331	41200	260	ETH-86780	40760	290	ETH-86779	38140	240
ETH-71322/MAMS	41220	210	ETH-71316	40790	250	ETH-86785 Layer I/J	39570	260
ETH-71315/MAMS	41310	180						
ETH-71320	41450	270						
ETH-71325	41480	270						
ETH-86784	41660	320						
ETH-71329	41730	280						
ETH-71314	41770	210						
ETH-71324/MAMS-28681*	41820	250						
ETH-86770 HS	41850	480						
ETH-71328	41850	280						
ETH-71323/MAMS-28680*	41950	250						
ETH-71330	42270	300						
ETH-86772	42450	510						
ETH-86786 Layer I/J	41740	320						
mean	41647			40623			39293	
stdev	374			178			669	
error of mean	94			89			334	
**P value 1 vs. 3**	**2,6E-08**		**P value 1 vs. 2**	**6,7E-05**		**P value 2 vs. 3**	**0,01584**	

In addition, we used the Kernel Density Estimation (KDE) in combination with the Bayesian model within the OxCal software (v4.4) [63] to objectively see the length distributions of each phase based on the radiocarbon dates we have (S3 Fig and S1–S3 Texts).

This situation leads us to conclude that layer N1-I could be composed of a different temporal cluster of dates, hence different human occupation/palimpsests, which were not recognised by either the geomorphological or lithic studies due to the low sedimentation rate that compressed the human occupations into a 5–8 cm thick layer that thins towards the cave walls [35] (page 2 in the SI of [35]).

#### 3 Phases model

To build the 3 phases model, we applied a three contiguous Phase model in OxCal, assuming that one phase follows another.

In the 3 phases model, we used a total of 25 dates, including more ‘prior’ information. We divided the N1-I layer into three stages based on both the t-test and the KDE in combination with the Bayesian model: Group 1 with 17 dates, including 16 from layer N1-I around 41,000 ^14^C BP [35] and one from layer N1-I/J (ETH-86786 published in S2 Table in [35], but excluded from the model since was identified as outliers in the Bayesian model). The second group has four dates, all from the N1-I layer, with a ^14^C age of around 40,000 ^14^C BP [35]. The third group is formed by four more dates, all published in S2 Table in [35]. Three are from N1-I, the human bone ETH-86769 and ETH-86782 and ETH-86779 and one from N1-I/J. All except the human bone ETH-86769 were excluded from the model since they were identified as outliers in the Bayesian model. The group has ^14^C ages around 39,000 ^14^C BP. Each phase is separated by a transitional boundary.

#### 2 Phases model

The youngest dates (circa 39,000 to 38,000 ^14^C BP) could be all outliers, as indicated by the published model in [35], since they do not reflect the consistency of the other 20 radiocarbon dates. Following this situation, we built an extra model where we considered only two distinct phases in the same layer, N1-I. the separation is based on both the t-test and the KDE in combination with the Bayesian model as the 3 phases model.

To build the 2 phases model, we applied a two contiguous Phase model in OxCal, assuming that one phase is following another.

The ‘date’ command, both in the 3 phases and 2 phases model in OxCal, was used to establish not only the probability distribution functions (PDFs) ranges, e.g., start and end boundaries of archaeological divisions and different geological layers, but also to estimate the duration of the IUP.

## Results

### ^14^C error range and Kauri floating curve

The calibrated ranges of eight direct radiocarbon dates of hominin are placed in the different parts of the calibration curve, five in IntCal20 (the oldest part) and three with the IntCal20+Kauri.

Direct dates of two Neanderthals (Vindija 33.19 and Fonds-de-Forêt) were obtained with the HYP [6, 22]. Six dates were obtained with the standard ultrafiltration protocol [35, 38, 58, 59] (Table 1 and Fig 1). Four direct dates of *Homo sapiens* from the Bacho Kiro site [35, 64] are obtained with a small ^14^C error range. Two more are the direct dates of Neanderthals from the Vindija site in Croatia, with smaller ^14^C errors than the one produced by the HYP on the same specimen [59] (Table 1 and Fig 1).

The comparison between the direct radiocarbon dates by using a high-resolution approach and the HYP, a method that generally delivers a larger ^14^C error, shows that the calibrated ranges of the specimens of Vindija 33.19 and Fonds-de-Forêt are very large (Fig 1B and 1C), even at 68.3% probability. In fact, Vindija 33.19, with an error of ±1200 years, is placed between the cold GS13 and the warm GI12 stages in comparison with the two overlapping calibrated ranges obtained on different bones but from the same Neanderthal site (Vindija 33.26 and 33.39, with a ^14^C error of ±550 and ±600 years respectively). These latest dates place the Neanderthals from Vindija, in Croatia, during the very end of the cold GS13 and mostly during the warm GI12 event.

On the other part of the curve where Kauri trees play their role, the Fonds-de-Forêt fossil has a calibrated range that includes two of the four Bacho Kiro dates (ETH-86771 and ETH-86769) and, even in this case, it is difficult to distinguish if this Neanderthal from Belgium was living during the last part of the cold event GS12 or during the warm GI11.

The potential of having the Kauri data in this period is that we can use various probability distributions and not only the 68.3% range (Fig 1B and 1D). A clear example is given by the highly precise dates of Bacho Kiro and the combination with the new study of δ^18^O [65], which show that the four humans span three different climatic conditions starting from 45,440 to 42,700 cal BP (68.3%) (Table 1). On the other hand, in the new study on the δ^18^O of 13 faunal teeth [65], 12 of them are from the same layer as our dates (N1- [65]I), shows that the human occupation at Bacho Kiro should have occurred during a cold phase. This new information lets us focus only on sample ETH 86771, which has three of five probability ranges (at 68.3%) provided by the new Kauri section (the highest at 26.9%, followed by 17%, and another at 16.8%), that fall in the cold phase of GS12 (Fig 1D and Table 3).

**Table 3:** Radiocarbon ages of the direct dates of 4 *Homo sapiens* from Bacho Kiro and 1 Neanderthal from Fond-de Forêt. The respective calibrated results using IntCa20+Kauri in OxCal4.4 [58], the ranges obtained in the model of [35] calibrated using IntCal 20+Kauri section. The ranges obtained in the 3 phases model, calibrated using IntCal 20+Kauri section, and the ranges obtained in the 2 phases model, calibrated using IntCal 20+Kauri section.

Humans directly dated	Calibrated dates with Kauri		Modelled dates with Kauri in Fewlass et al. 2020		Modelled dates 3 Phases with Kauri in this paper				Modelled dates 2 Phases with Kauri in this paper			
	**From-to**	**68.3%**	**From-to**	**68.3%**	**From-to**	**68.3%**	**From-to**	**95.4%**	**From-to**	**68.3%**	**From-to**	**95.4%**
**ETH-86772 (42450;510)**	45440–44640	68.3%	44770–44460	66.8%	44650–44430	68.3%	44820–44340	95.4%	44650–44430	68.3%	44810–44340	95.4%
			43980–43960	1.4%								
												
**ETH-86770 (41850;480)**	45040–44310	65.4%	44720–44320	65.6%	44620–44400	68.3%	44770–44280	95.4%	44650–44400	68.3%	44760–44280	95.4%
	43980–43940	2.9%	43980–43950	2.7%								
** **												
**ETH-86771 (40600;420)**	43690–43360	26.9%	44370–43970	68.3%	44210–43970	25.4%	44350–43220%	95.4%	44310–43970	50.6%	44380–43620	95.4%
	43930–43700	17%			43940–43700	24.0%			43950–43790	13.9%		
	44190–43970	16.8%			43690–43550	13.1%						
	43340–43250	6.6%			43540–43490	3.2%			43770–43710	3.8%		
	43160–43130	1%			43460–43420	2.6%						
** **												
**ETH-86769 (39750;380)**	43260–42780	60.9%	44270–43970	64%	43110–42740	66.2%	43290–42610	90.1%	not Modelled	not Modelled		
	43380–43320	4.9%	43830–43810	2.8%	42720–42700	2%	43390–43300	3.1%	not Modelled	not Modelled		
	42770–42740	1.5%	43750–43730	1.5%			42560–42530	1%	not Modelled	not Modelled		
	42720–42700	1%					42470–42440	0.8%	not Modelled	not Modelled		
							42500–42480	0.2%				
							42420–42400	0.2%				
** **												
**OxA-38322 (39500;1100)**	43530–42390	57.6%							
	43670–43560	3.9%										
	44040–43970	1.9%										
	43760–43710	1.8%										
	43840–43790	1.5%										
	42350–42310	1.3%										
	42380–42360	0.3%										

Therefore, not having more prior information to constrain ranges of the other samples, it is difficult to discuss the temporal overlap between the same or two different human species, and their interaction with the climate, and it becomes even more difficult if the ^14^C error range is not so refined as the one from Bacho Kiro.

In fact, the calibrated age range of the Neanderthal of Fonds-de-Forêt in Belgium is 1,730 years (OxA-38322 ^14^C Age 39,500±1100 Tables 1 and 3), and it embeds chronologically two *Homo sapiens* from Bacho Kiro in Bulgaria (ETH-86769^14^C Age 39,750±380 span of 680 years, and ETH-86771 ^14^C Age 40,600±420 span of 1,060 years) (Table 1). Therefore, the use of two different ranges of ^14^C error in models (e.g., [66]) for claiming the temporal overlapping of the two species in two different regions should be viewed with some caution.

### Unmodelled vs Modelled of the direct dates of Bacho Kiro *Homo sapiens*

In S5 Table of the SI of [35], 21 dates were modelled in Niche Layer I (N1-I) and four dates in layer J (N1-J). Two more dates in layer N1-I (ETH-86782 and ETH-86779) were excluded from the model since they were identified as outliers from the Bayesian model. In addition, two more ^14^C dates were produced but never inserted in the Bayesian model of [35] because the bone samples were found at the interface between layers N1-I and N1-J.

For our exercise here, we will discuss two different Bayesian models in OxCal, using the Kauri section: 1) a 3 phases model in which we will include the two dates previously omitted in [35]; 2) a 2 phases model in which we also discard the date of ETH-86769 based on both the t-test and the KDE in combination with Bayesian model. Moreover, it is visible that in the Bayesian model of [35] (Fig 3B in [35]), this sample (ETH-86769) was forced towards the older calibrated ages resulting in a poor agreement of 12.7%, well below 60%.

Here we compare the calibrated ranges of the four direct radiocarbon dates of the human fossils of Bacho Kiro, found only in the Niche N1-I layer already shown and discussed above with the modelled ages produced in [35] and the two new Bayesian models built for this paper (S1 File and Fig 2). The OxCal model codes are given in the SI).

- Modelled dates in [35]

The results obtained from the modelled ages in the publication of [35] are quite different from the unmodelled ranges (Fig 2B and 2C). In fact, in the Bayesian model of [35] all the dates are constrained towards the older part of the calibration curve. This artificial constraint for the youngest and the oldest ages of the *Homo sapiens* is due to the fact that more than half of the dates are statistically robust around 41,000 cal BP and OxCal assuming a uniform distribution. If we assume that this is correct, then the human date ETH-86772 modelled with IntCal20+Kauri is 44,700–44,460 cal BP [(66.8%), which implies that this human was living during the warm part of the GI12 (Fig 2C, Table 3). However, there is 1.4% probability that this hominin was living between 43,980–43,960 cal BP which is during the cold phase of GS12. The ETH-86770 human modelled age range is overlapping in time with ETH-86772 for both the 65.6% and 2.6% probability (Fig 2C, Table 3). For ETH-86771, the entire 68.3% age range, which was mostly in the cold period for the IntCal20 calibrated ranges now (Fig 2C, Table 3) extends to the end of the warm phase GI12, while the major part falls in the cold phase of GS12 overlapping with the ETH-86769.

- Modelled dates in 3 phases model

While the ranges of the four humans obtained with the model of [35] (Fig 2C) are more constrained, the results obtained with our new 3 phases model are broader (Fig 2D green bars), and more close to the unmodelled dates (Fig 2A). Moreover, they are temporally quite separate from each other, demonstrating that the *Homo sapiens* could have been at the site in three different time periods. Two of them (ETH-86772 and ETH-86770) possibly occupied the site between 44,650 to 44,400 cal BP in a warm phase of the GI12. Then, about 190 to 390 years later, the specimen ETH-86771 inhabited the cave during the cold climatic condition of GS12. After at least 310 years, the specimen ETH-86769 settled at the site at the beginning of warm interstadial GI11.

- Modelled dates in 2 phases model

In the 2 phases model (Fig 2D yellow bars), we considered only two distinct phases in the same layer N1-I, Group 1 and 2, with all the youngest ages as outliers, including the ETH-86769. The ranges of the samples ETH-86772 and ETH-86770 remain unvaried, whereas the range of fossil ETH-86771 is shorter and falls almost entirely at the beginning of GS12 (Fig 2D, yellow bars).

### Unmodelled vs Modelled IUP Boundaries of Bacho Kiro site

We add the ‘date’ command in OxCal to infer the duration of the IUP, layer N1-I, at the site (S1 File and Fig 3) to the model of [35] and the new models we built.

If we consider only the calibrated dates for the start and the end of the IUP, the IUP spans 2,740 years, from 45,440 to 42,700 cal BP. This long range includes several warm and cold phases. On the contrary, the modelled ranges in [35] demonstrate that the range for the IUP is very short, only 600 years at the end of the warm phase (GI12) before the beginning of the Greenland cold stadial GS12, spanning between 44,670 to 44,070 cal BP.

In the case of the 3 phases model, the range of the IUP is between 44,490 to 42,780 cal BP for a total of 1,710 years, 1,030 years less than the range obtained from the calibrated dates, and 1,110 years more than the range obtained in [35]. In the case of the 2 phases model, the range of the IUP is between 44,630 to 43,860 cal BP (68.3%) for a total of 770 years, spanning 170 years longer than the interval obtained in [35].

## Discussion

Understanding the timing of dispersals of *Homo sapiens* in Europe, the interaction with Neanderthals, and the extent of their temporal overlapping in the different regions is a research topic that requires high accuracy and precision in radiocarbon dating. Here, by using the high-resolution chronology of the site of Bacho Kiro, we have demonstrated the potential and the benefit that the interplay between the advanced bone pretreatment method, the advanced MICADAS AMS system, the small ^14^C error range, and the integration of the new Kauri glacial floating tree-ring datasets in IntCal could generate. The widespread application of the radiocarbon 3.0 approach to the European key sites in this timeframe could revolutionize the current scenarios and explain the unsolved questions in this important period of our human history.

The comparison between chronometric data with wide and small ^14^C error ranges has shown the advantages that the latter could deliver in terms of temporal and environmental precision (Figs 1, 2). Although the HYP is a helpful method to elucidate approximately the calibrated range of human fossils or artefacts when the sample is heavily contaminated by glue, its chronological resolution is too low for debating the disappearance of Neanderthals or discussing the influence of climatic conditions on their demise. The other claim that the HYP is presumably the only method that could produce more accurate, robust results [6, 22, 67] is also debatable because we demonstrated that an accurate bone pretreatment using the ultra-filtration could achieve analogous and more precise radiocarbon dates (Fig 1B, 1C). In general, the wide ranges delivered by HYP, OSL, TL or ESR methods and any ^14^C date with an error range higher than 500 years for this time period on human bones, is too inaccurate for solving the dispute on when Neanderthals and *Homo sapiens* interacted, and should be used cautiously.

We are aware that the integration of new glacial tree-ring datasets in IntCal is so far limited. However, the small ^14^C error range in the radiocarbon dates could still make a great difference. In Fig 1B and 1C, we demonstrated the higher precision of the samples Vindija 33.26 and 33.39 in comparison with the sample 33.19 OxA-HYP once calibrated in IntCal20. Therefore, the use of ranges of the latter should be avoided from now on for debating the disappearance of Neanderthals in Croatia.

Moreover, even though the radiocarbon community has accepted the discussion of calibrated ranges at 95.4%, we have shown that with the use of the small ^14^C error range and the advanced calibration curve (IntCal+Kauri), even spatiotemporal archaeological discussion can be brightly addressed using only the 68.3%, and the range overlapping at the 95.4% probability should be considered a maximum probable overlap.

Last but not least, the scope of the integration of the new glacial tree-ring datasets in IntCal and the further subdivision of the 68.3% probability has also great potential. As it has been shown by using the Kauri curve, the ranges of the human fossils of Bacho Kiro are additionally sectioned into different intervals. Cross-referencing with the, e.g., δ^18^O in tooth enamel and the ice core data to explore which one of these intervals is the most likely could detail deeper an already high-resolution chronology, inferring more precisely the climatic and environmental changes and disclosing temporal palimpsests that were not recognized in the first publication.

The previously proposed study of Bacho Kiro chronology placed the end of the IUP at 44,130 cal BP (using IntCal20), and in this paper, it is at 44,070 cal BP (using the new Kauri), both at 68.3%. However, the duration of the IUP in Europe is mostly based on the sequence of Bacho Kiro because of the uncertainty of the chronology of the other IUP sites (see references in [7]) and the lack of sedimentary archives in which a clear succession between IUP and Protoaurignacian is found.

The ranges of the IUP published in [35] show how the Bayesian model (assuming uniform distribution) can constrain too much the dating of an archaeological layer because more than half of the dates are around 41,000 ^14^C BP. This strong constraint does not reflect the long-range produced using only the calibrated dates (2740 years vs 600 years of the model) (Fig 3B and 3C). Our new models, with the three or two different phases, show a more convincing scenario spanning the IUP respectively for 1710 years or 770 years (Fig 3D, S1 File) in comparison with the model of [35].

In this study, we verified 2 possible scenarios of the end of the IUP, adding and omitting in our Bayesian models the younger dates of Group 3. The 3 phases model indicates the end of the IUP at 42,780 cal BP at 68.3%, while the 2 phases model infers an age at 43,860 cal BP at 68.3% (S1 File, Fig 3C and 3D). At the moment, both scenarios could be supported since it is still unknown if the IUP could have lasted longer in Bacho Kiro in comparison with the Levant or could have temporally overlapped with the dispersal of the Protoaurignacian. However, it is worth noticing that the crossing of the chronometric data with the Paleogenetic studies [64, 68] makes the younger age of ETH-86769 suspicious because the hominin analysed with the mDNA study is among the oldest *Homo sapiens* in the genetic tree (Fig 2 of [64]). On the other hand, there are three more dates that have the same range as the ETH-86769 specimen. This situation implies that greater interaction between the different disciplines is needed for unveiling which model is more correct since adding or omitting a set of dates could produce different scenarios, as well as knowing more information (e.g., δ^18^O study) will make the interpretation of the archaeological site more valuable.

In fact, the dental δ^18^O study on the 13 samples analysed, 12 from the Niche and one from the main sector, from the IUP layer of Bacho Kiro [65] shows that the IUP, hence the human occupation at the site, happened during cold climate conditions (GS12). The conclusion of the study was not in accordance with the boundary of the IUP created by the chronological model built in [35], concluding that chronometric correlations between archaeological deposits and climate records are not always appropriate since the resolutions of the different datasets are not high enough. Moreover, given the lack of refined radiocarbon dates for other European IUP sites, it is still challenging to robustly support alternative climatic models.

Our example using Bacho Kiro as the only site with more than 21 high precision radiocarbon dates for one single layer shows that we can forecast possible scenarios of human/environment dynamics during climatic fluctuations. In fact, in our 3 phases model, we demonstrate that the fossils ETH-86772 and ETH-86770 settled the site during GI12, the specimen ETH-86771 during the cold GS12, and ETH-86769 at the beginning of GI11 (Fig 2D). In the 2 phases model, the results do not change with only the fossil ETH-86771 facing the climatic deterioration of GI12 (Fig 2D). In this case, we demonstrate that using radiocarbon 3.0 it is possible to correlate archaeological deposits with climatic records in an accurate way.

## Conclusion

By exploring high-resolution chronological sequences and key Neanderthals/*Homo sapiens* sites in Europe, the field of archaeology, archaeometry, and landscape archaeology will draw key specimens into more comprehensive regional and European models, which have broad implications for understanding large-scale movements and developments through human history.

Our exercise shows that using radiocarbon 3.0 (updated radiocarbon pretreatment, the latest AMS instrumental advances, and the application of the Bayesian model coupled with the new IntCal20, including the Kauri floating tree-ring section), we are able to accomplish the definitive high resolution of European key archaeological sites during recurrent climate fluctuations, and model the human and faunal species’ responses from a diachronic perspective. In this way, we will promote knowledge exchange between archaeology, palaeoclimatology, geochronology, and geosciences in general, all essential disciplines in the study of the human past.

High-resolution radiocarbon analysis on a single layer of a site, such as that presented here, offers additional insights into major European archaeological sites and facilitates understanding of the human occupation at the site and the resilience of hominins to the different climatic fluctuations that possibly contributed to the expansion of *Homo sapiens* around the world.

## Supporting information

S1 File Radiocarbon dates from layers J and I of the Niche of Bacho Kiro site published in [35], as well as the direct dates of human fossils in red. In light blue are the calibrated dates (Unmodelled and Modelled) of the Niche published in [35] using only IntCal20. In green are the calibrated dates (Unmodelled and Modelled) of the Niche published in [35] using IntCal20+the Kauri section. In orange, the 3 phases model calibrated dates (Unmodelled and Modelled) of the Niche published in [35] using IntCal20+the Kauri section. In orange, the 2 phases model calibrated dates (Unmodelled and Modelled) of the Niche published in [35] using IntCal20+the Kauri section.
(XLSX)

S1 Fig The calibrated ranges of the 4 Bacho Kiro *Homo sapiens in 3 phases model*. The calibrated ranges both at 68.3% and at 95.4% of the 4 Bacho Kiro *Homo sapiens* directly dated obtained using the 3 phases model (light green).
(DOCX)

S2 Fig The calibrated ranges of the 4 Bacho Kiro *Homo sapiens in 2 phases model*. The calibrated ranges both at 68.3% and at 95.4% of the 4 Bacho Kiro *Homo sapiens* directly dated obtained using the 2 phases model (dark yellow).
(DOCX)

S3 Fig Kernel Density Estimation plots (KDE) for the different layers of Bacho Kiro. Estimation of the distribution of each layer (N1-J and N1-I) and sub-layers of N1-I of Bacho Kiro and Bayesian start (green)/end (red) date boundaries.
(DOCX)

S1 Text The CQL codes from OxCal 3 phases model. (DOCX)

S2 Text The CQL codes from OxCal 2 phases model. (DOCX)

S3 Text The CQL code from OxCal 3 phases model with Kernel Density Estimation (KDE). (DOCX)
