# Correction: Back to the future: The advantage of studying key events in human evolution using a new high resolution radiocarbon method

**DOI:** 10.1371/journal.pone.0346362

**Published:** 2026-03-31

**Authors:** Sahra Talamo, Bernd Kromer, Michael P. Richards, Lukas Wacker

The following information is missing from the Funding statement: S.T. is funded by FARE (Framework per l’attrazione ed il rafforzamento delle eccellenze per la Ricerca in Italia – III edizione), ‘Evaluate the precision of time in Human Evolution adopting spectrometric methods for archaeological bones – EURHOPE’, Prot. R20L4N7MS5, CUP J53C2200374000.
