# Supplementary material for: Back to the future: The advantage of studying key events in human evolution using a new high resolution radiocarbon method
Source: PLoS One. 2023 Feb 15;18(2):e0280598. doi: 10.1371/journal.pone.0280598 (PMC9931112; doi:10.1371/journal.pone.0280598)
Supplement: S1 Fig — The calibrated ranges both at 68.3% and at 95.4% of the 4 Bacho Kiro Homo sapiens directly dated obtained using the 3 phases model (light green). (DOCX) [file pone.0280598.s002.docx]

**SUPPORTING INFORMATION**

**Back to the future: the advantage of studying key events in human evolution using a new high resolution radiocarbon method.**

Sahra Talamo, Bernd Kromer, Michael P. Richards, Lukas Wacker


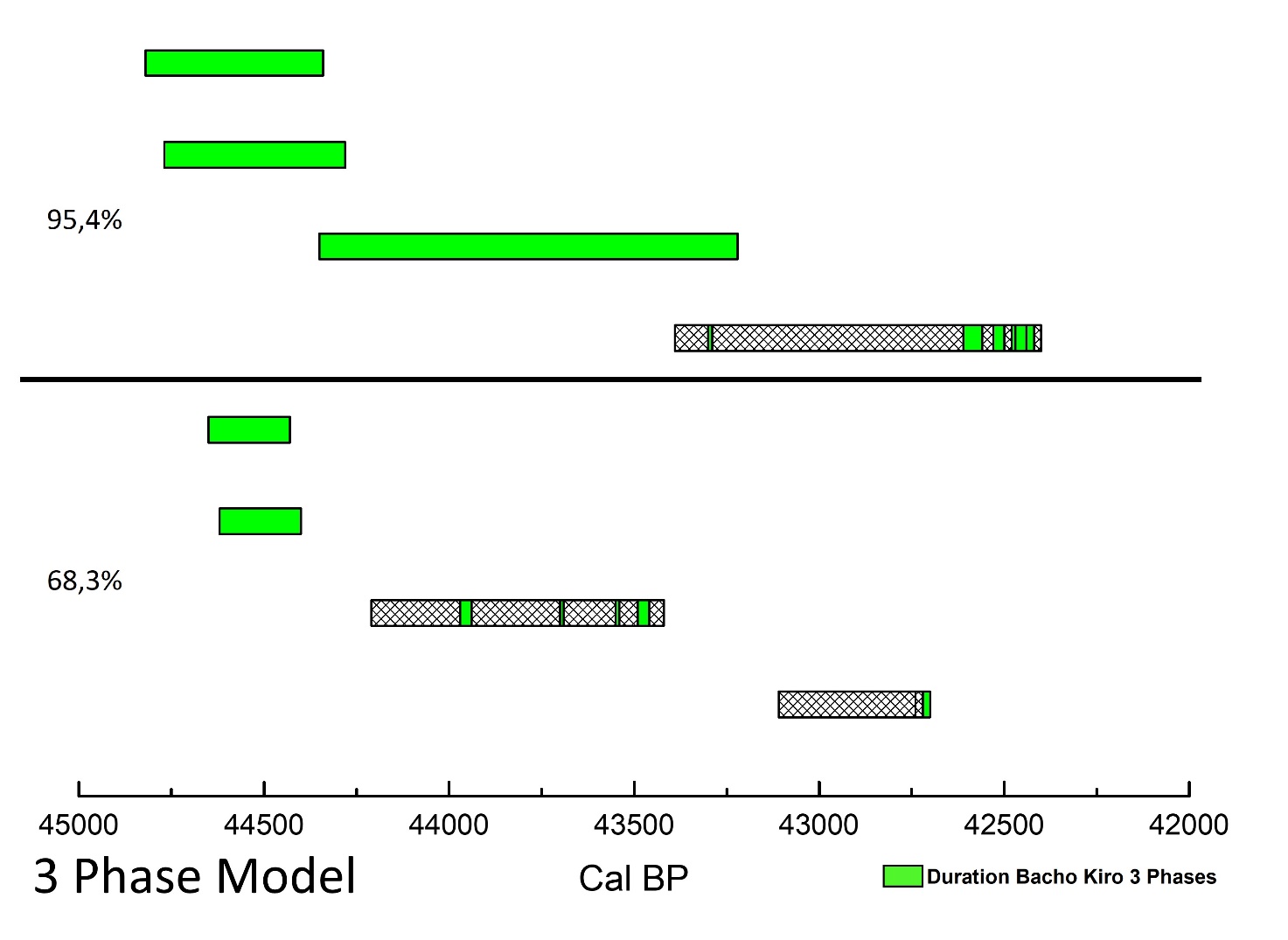


**S1 Fig.** **The calibrated ranges of the 4 Bacho Kiro *Homo sapiens*** ***in 3 phases model*.** The calibrated ranges both at 68.3% and at 95.4% of the 4 Bacho Kiro *Homo sapiens* directly dated obtained using the 3 phases model (light green).
