# Supplementary material for: Back to the future: The advantage of studying key events in human evolution using a new high resolution radiocarbon method
Source: PLoS One. 2023 Feb 15;18(2):e0280598. doi: 10.1371/journal.pone.0280598 (PMC9931112; doi:10.1371/journal.pone.0280598)
Supplement: S2 Fig — The calibrated ranges both at 68.3% and at 95.4% of the 4 Bacho Kiro Homo sapiens directly dated obtained using the 2 phases model (dark yellow). (DOCX) [file pone.0280598.s003.docx]

**SUPPORTING INFORMATION**

**Back to the future: the advantage of studying key events in human evolution using a new high resolution radiocarbon method.**

Sahra Talamo, Bernd Kromer, Michael P. Richards, Lukas Wacker


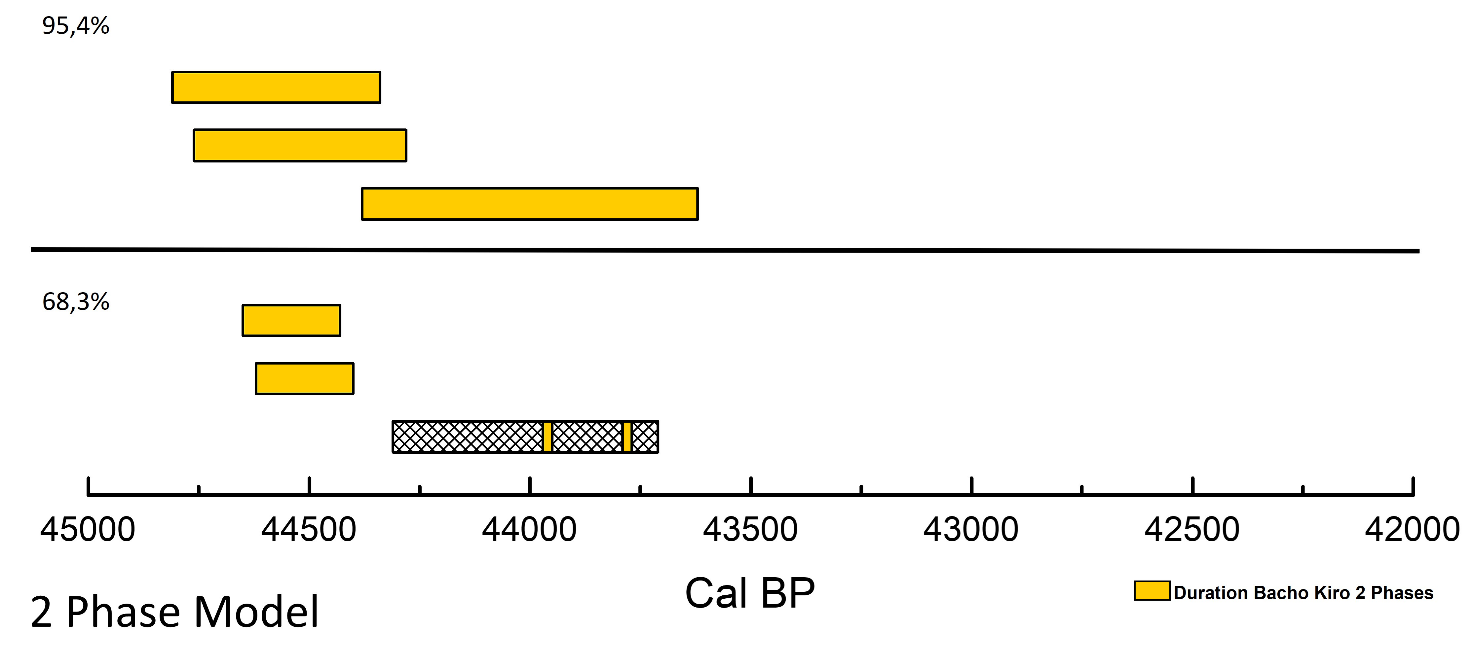


**S2 Fig. The calibrated ranges of the 4 Bacho Kiro *Homo sapiens in 2 phases model*.** The calibrated ranges both at 68.3% and at 95.4% of the 4 Bacho Kiro *Homo sapiens* directly dated obtained using the 2 phases model (dark yellow).
