# Supplementary material for: Back to the future: The advantage of studying key events in human evolution using a new high resolution radiocarbon method
Source: PLoS One. 2023 Feb 15;18(2):e0280598. doi: 10.1371/journal.pone.0280598 (PMC9931112; doi:10.1371/journal.pone.0280598)
Supplement: S3 Fig — Estimation of the distribution of each layer (N1-J and N1-I) and sub-layers of N1-I of Bacho Kiro and Bayesian start (green)/end (red) date boundaries. (DOCX) [file pone.0280598.s004.docx]

**SUPPORTING INFORMATION**

**Back to the future: the advantage of studying key events in human evolution using a new high resolution radiocarbon method.**

Sahra Talamo, Bernd Kromer, Michael P. Richards, Lukas Wacker

**
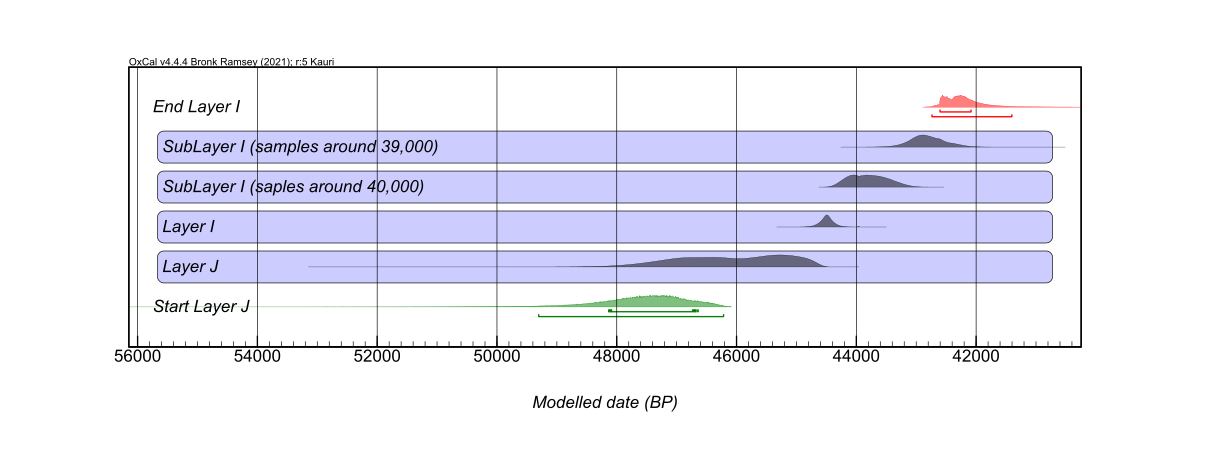
**

**S3 Fig**. **Kernel Density Estimation plots (KDE) for the different layers of Bacho Kiro.** Estimation of the distribution of each layer (N1-J and N1-I) and sub-layers of N1-I of Bacho Kiro and Bayesian start (green)/end (red) date boundaries.
