# Supplementary material for: Back to the future: The advantage of studying key events in human evolution using a new high resolution radiocarbon method
Source: PLoS One. 2023 Feb 15;18(2):e0280598. doi: 10.1371/journal.pone.0280598 (PMC9931112; doi:10.1371/journal.pone.0280598)
Supplement: S1 Text — (DOCX) [file pone.0280598.s005.docx]

**SUPPORTING INFORMATION**

**Back to the future: the advantage of studying key events in human evolution using a new high resolution radiocarbon method.**

Sahra Talamo, Bernd Kromer, Michael P. Richards, Lukas Wacker

**S1 Text. CQL Code from OxCal 3 Phases Model**

Plot()

{

Curve("intcal20Kauri40k.14c");

Sequence("Niche 1, Bacho Kiro Cave")

{

Boundary("Start Layer J");

Phase("Layer J")

{

R_Date("ETH-93196", 45120, 490)

{

color="green ";

};

R_Date("ETH-86787*", 44890, 450)

{

color="green ";

};

R_Date("ETH-86789*", 42900, 370)

{

color="green ";

};

R_Date("ETH-93194", 42670, 370)

{

color="green ";

};

};

Boundary("Transition Layer J/Layer I");

Phase("Layer I")

{

R_Date("ETH-86772", 42450, 510)

{

color="purple";

};

R_Date("ETH-71330*", 42270, 300)

{

color="green";

};

R_Date("ETH-71323/MAMS-28680*", 41950, 250)

{

color="green";

};

R_Date("ETH-71328*", 41850, 280)

{

color="green";

};

R_Date("ETH-86770", 41850, 480)

{

color="purple";

};

R_Date("ETH-71324/MAMS-28681*", 41820, 250)

{

color="green";

};

R_Date("ETH-71314*", 41770, 210)

{

color="green";

};

R_Date("ETH-86786", 41740, 320);

R_Date("ETH-71329*", 41730, 280)

{

color="green";

};

R_Date("ETH-86784*", 41660, 320)

{

color="green";

};

R_Date("ETH-71325*", 41480, 270)

{

color="green";

};

R_Date("ETH-71320*", 41450, 270)

{

color="green";

};

R_Date("ETH-71315/MAMS-28677*", 41310, 180)

{

color="green";

};

R_Date("ETH-71322/MAMS-29483*", 41220, 210)

{

color="green";

};

R_Date("ETH-71331*", 41200, 260)

{

color="green";

};

R_Date("ETH-71327*", 41170, 260)

{

color="green";

};

R_Date("ETH-71318*", 41080, 260)

{

color="green";

};

};

Boundary("Transition main layer I/Sublayer I40");

Phase("Sublayer I 40,000")

{

R_Date("ETH-71316*", 40790, 250)

{

color="green";

};

R_Date("ETH-86780*", 40760, 290)

{

color="green";

};

R_Date("ETH-86771", 40600, 420)

{

color="purple";

};

R_Date("ETH-86783*", 40340, 280)

{

color="green";

};

};

Boundary("Transition sublayer I40/Sublayer I39");

Phase("Sublayer I 39000")

{

R_Date("ETH-86769", 39750, 380)

{

color="purple";

};

R_Date("ETH-86782", 39710, 260);

R_Date("ETH-86785", 39570, 260);

R_Date("ETH-86779", 38140, 240);

};

Boundary("End Layer I");

};

Sequence()

{

Boundary("=Start Layer J");

Date("IUP");

Boundary("=End Layer I");

};

};
