# Supplementary material for: Back to the future: The advantage of studying key events in human evolution using a new high resolution radiocarbon method
Source: PLoS One. 2023 Feb 15;18(2):e0280598. doi: 10.1371/journal.pone.0280598 (PMC9931112; doi:10.1371/journal.pone.0280598)
Supplement: S3 Text — (DOCX) [file pone.0280598.s007.docx]

**SUPPORTING INFORMATION**

**Back to the future: the advantage of studying key events in human evolution using a new high resolution radiocarbon method.**

Sahra Talamo, Bernd Kromer, Michael P. Richards, Lukas Wacker

**S3 Text. CQL Code from OxCal 3 Phases Model with Kernel Density Estimation (KDE)**

Plot()

{

Curve("intcal20Kauri40k.14c");

Sequence()

{

Boundary("Start Layer J")

{

color="green";

};

Phase("Layer J")

{

KDE_Plot("Layer J")

{

};

Sum("Sum LayerJ")

{

};

R_Date("ETH-93196", 45120, 490);

R_Date("ETH-86787*", 44890, 450);

R_Date("ETH-86789*", 42900, 370);

R_Date("ETH-93194", 42670, 370);

};

Boundary("Transition Layer J/Layer I")

{

};

Phase("Layer I")

{

KDE_Plot("Layer I")

{

};

Sum("Sum LayerI")

{

};

R_Date("ETH-86772", 42450, 510);

R_Date("ETH-71330*", 42270, 300);

R_Date("ETH-71323/MAMS-28680*", 41950, 250);

R_Date("ETH-71328*", 41850, 280);

R_Date("ETH-86770", 41850, 480);

R_Date("ETH-71324/MAMS-28681*", 41820, 250);

R_Date("ETH-71314*", 41770, 210);

R_Date("ETH-86786", 41740, 320);

R_Date("ETH-71329*", 41730, 280);

R_Date("ETH-86784*", 41660, 320);

R_Date("ETH-71325*", 41480, 270);

R_Date("ETH-71320*", 41450, 270);

R_Date("ETH-71315/MAMS-28677*", 41310, 180);

R_Date("ETH-71322/MAMS-29483*", 41220, 210);

R_Date("ETH-71331*", 41200, 260);

R_Date("ETH-71327*", 41170, 260);

R_Date("ETH-71318*", 41080, 260);

};

Boundary("Transition Layer I/SubLayer I40")

{

color="red";

};

Phase("SubLayer I40")

{

KDE_Plot("SubLayer I40")

{

};

Sum("Sum SubLayerI40")

{

};

R_Date("ETH-71316*", 40790, 250);

R_Date("ETH-86780*", 40760, 290);

R_Date("ETH-86771", 40600, 420);

R_Date("ETH-86783*", 40340, 280);

};

Boundary("Transition SubLayer I40/SubLayer I39")

{

};

Phase("SubLayer I30")

{

KDE_Plot("SubLayer I39")

{

};

Sum("Sum SubLayerI39")

{

};

R_Date("ETH-86769", 39750, 380);

R_Date("ETH-86782", 39710, 260);

R_Date("ETH-86785", 39570, 260);

R_Date("ETH-86779", 38140, 240);

};

Boundary("End Layer I39")

{

color="red";

};

};

};
